# Web-Based Dissemination of a Civic Engagement Curriculum to Promote Healthy Eating and Active Living in Rural Towns: The eHEART Study

**DOI:** 10.3390/ijerph17072571

**Published:** 2020-04-09

**Authors:** Rebecca Seguin-Fowler, Meredith Graham, Urshila Sriram, Galen Eldridge, Jimin Kim, Madeleine Tom

**Affiliations:** 1 Texas A&M AgriLife Research, College Station, TX 77843, USA; us54@cornell.edu (U.S.); ge77@tamu.edu (G.E.); 2 Division of Nutritional Sciences, Cornell University, Ithaca, NY 14853, USA; mlg22@cornell.edu (M.G.); jk2586@cornell.edu (J.K.); mft44@cornell.edu (M.T.)

**Keywords:** rural health, food environment, built environment, civic engagement

## Abstract

Civic engagement interventions aimed at improving food and physical activity environments hold promise in addressing rural health disparities, but ensuring feasible and sustained dissemination remains a challenge. The present study aimed to evaluate the feasibility of a civic engagement curriculum adapted for online dissemination (Healthy Eating and Activity in Rural Towns (eHEART)). The eHEART curriculum and website were developed based on feedback from local health educators and community members. eHEART groups were facilitated by local Extension educators across three rural towns in three U.S. states (Montana, Wisconsin, and Alaska). Implementation feasibility was assessed through monthly project reports and interviews with educators. All eHEART groups successfully completed curriculum activities and met their project goals after nine months (November 2018 to July 2019). Groups ranged in size from 4 to 8 community residents and implemented varied strategies to improve aspects of their local food and/or physical activity environments. Facilitators of implementation included clear guidance on facilitating curriculum activities and the flexible and community-driven nature of eHEART projects. Recommended changes included more guidance on evaluating projects and contacting stakeholders as well as providing online tools and support for project management. Findings from this work have important implications for creating healthier rural environments. Local health educators and other community groups can feasibly use the eHEART curriculum to foster environmental changes that support healthy eating and active living.

## 1. Introduction

Rural populations in the United States experience higher rates of obesity and are less likely to engage in health-promoting behaviors (e.g., adequate diet, physical activity) than urban populations [1,2]. These disparities are partly due to environmental constraints in rural areas, including limited access to nutritious foods and opportunities for physical activity [3,4]. Creating environmental change through civic engagement is one promising strategy for improving access to food and physical activity resources. Existing studies suggest that engaging local residents can lead to meaningful built environment and policy changes in urban and rural settings [5,6,7,8,9,10]. 

Guided by community-based participatory research methods, the Healthy Eating and Activity in Rural Towns (HEART) Club civic engagement curriculum provides a step-wise process for rural residents to improve healthy eating and physical activity opportunities in their community. The HEART Club program (also known as the Change Club) was first developed, implemented, and evaluated in the period 2010–2011 [7]. Several follow-up studies refining the materials and process have been conducted since then, with successful achievement of goal setting and attainment [8,9]. 

To improve the program’s reach, adoption, and effectiveness, the HEART Club curriculum was adapted into a web-based format (eHEART) and evaluated with Extension partners in three states. The present study describes the implementation and feasibility of the eHEART approach. 

## 2. Materials and Methods 

### 2.1. HEART Club Program Curriculum

The HEART Club curriculum consists of four structured 90 min meetings, ideally held one week apart, with additional action items to be completed between meetings. Groups are encouraged to schedule regular meetings after completing curriculum activities to achieve project goals. During curriculum meetings, Extension educators guide group members through the process of assessing, prioritizing, planning, and implementing environmental changes in their community. HEART Clubs begin by conducting a walkabout to identify barriers and assets to healthy eating and active living in their community. After reflecting on the walkabout findings, members identify an issue related to the local food or physical activity environment to work towards. Groups then establish specific, measurable benchmarks to address their chosen issue, including (1) choosing a strategy, (2) identifying stakeholders, (3) pilot testing and implementing, (4) monitoring and evaluation, and (5) planning for expansion.

### 2.2. eHEART Curriculum and Website Development

Adaptations to the HEART Club curriculum for web-based dissemination were informed by focus groups and interviews with former HEART Club members and facilitators. Qualitative data were descriptively coded to identify positive and negative aspects of the program curriculum, and recommendations for improvement. Notable curriculum changes included providing example community projects, restructuring the community walkabout, and offering additional guidance on contacting stakeholders and continuing group meetings. Table 1 provides an outline of eHEART curriculum activities. 

Research team members worked with a local web developer to create an online dissemination platform using Drupal (an open-source content management system). This website was designed to guide users through the eHEART curriculum and could be used to directly facilitate curriculum meetings or as a training tool. The responsive interface allows users to access the eHEART website via computer, tablet, or mobile phone. The homepage provides an overview of the eHEART process, instructions on navigating the website, and application guidelines for community groups. Meeting pages feature whiteboard videos and brief written descriptions of curriculum activities with links to handouts and worksheets. Full PDF versions of the eHEART curriculum are also available for users to download and use for facilitation. Resource pages include information on previous HEART Club projects (videos and written summaries), identifying stakeholders, and effective leadership skills. 

Telephone feedback sessions were held with twelve Extension educators from five states to evaluate the functionality and design of the eHEART website. Overall feedback was positive, with educators describing the website as clear and easy to navigate. Recommended changes included streamlining content on the homepage, reorganizing website tabs, adding more project examples, and providing information on applying for the eHEART program. These suggestions were discussed by the research team and informed website revisions. Components of the eHEART website are described in Table 2 (see Supplementary Figure S1 for screenshots of each webpage).

### 2.3. eHEART Application Process

To assess the feasibility of the eHEART approach, Extension educators from seven states were invited to disseminate the eHEART website and application form to local community organizations and groups (e.g., parent-teacher associations, playground committees). Educators were affiliated with other evidence-based programs implemented by Seguin and colleagues [11]. 

Groups were encouraged to apply for the eHEART program if they were interested in improving access to healthy food or physical activity opportunities in their community. Applicants were selected based on alignment with eHEART program objectives and were provided with $1000 to implement their community projects. Selected groups were given time to recruit additional members before starting the eHEART process. Designated leaders for each eHEART group were asked to review the website and complete an online training session prior to facilitating curriculum meetings. Leaders were also given a detailed timeline of project deliverables (i.e., collecting baseline surveys, scheduling curriculum meetings, and submitting progress reports). Supplementary Figure S2 outlines the specific deliverables and corresponding timelines. 

### 2.4. Data Sources and Analysis

Participants completed an online demographic questionnaire prior to starting eHEART meetings. To monitor group progress, eHEART leaders were asked to submit monthly project reports after completing all curriculum activities. Reports were submitted online through Qualtrics’ survey platform (Qualtrics, Provo, UT, USA). Interviews were conducted with leaders following the completion of group projects to assess satisfaction with the eHEART website and program facilitation. All participants provided written informed consent upon joining the eHEART study and oral consent was obtained prior to recording the interviews. All study procedures and materials were approved by Cornell University’s Institutional Review Board (IRB).

Participant characteristics were summarized using means for continuous variables and frequencies for categorical variables. Progress reports were used to document project activities, meeting frequency, and attendance levels. Qualitative interview responses were categorized as follows: positive aspects, challenging aspects, and recommendations for improvement. All analyses were conducted in 2019.

## 3. Results

Nine groups completed eHEART applications in the summer of 2018. Two groups that submitted applications were disqualified based on proposed group activities not meeting program objectives; one could not be reached for follow up; and three groups withdrew upon further review of the project timeline and activities. The three remaining groups successfully completed eHEART curriculum activities and initiated community projects between November 2018 and July 2019. All eHEART groups were led by Extension educators and took 2 to 4 months to complete the prescribed meetings and 8 to 9 months to complete their projects.

Table 3 presents demographic characteristics for two eHEART groups. The third group consisted primarily of secondary school students and the study IRB protocol only permitted data collection for adults.

Table 4 provides a summary of eHEART project activities across the three study towns. Town 1, Montana, has a population of approximately 3800 people (42% White, 46% American Indian/Alaska Native, 11% Latino or Hispanic); 20% of residents live in poverty [12]. Town 1 aimed to increase awareness of community trails among local residents and promote physical activity through trail use. The group collaborated with a local health coalition to develop signage and brochures highlighting existing trail routes. See Supplementary Figure S3 for trail brochures.

Town 2, Wisconsin, has a population of approximately 2200 people (87% White, 6% Latino or Hispanic); 9% of residents live in poverty [13]. Town 2 aimed to improve the physical activity and food environment at a local middle school. The group brainstormed ways to enhance indoor recess activities and outdoor play areas. They also advocated for changing the cafeteria layout to encourage healthier food choices and scheduling recess before lunch, which has been shown to improve dietary intake and classroom behaviors [14,15,16]. See Supplementary Figure S4 for photos of indoor and outdoor recess projects.

Town 3, Alaska, has approximately 4700 people (82% White, 8% American Indian/Alaska Native); 13% of residents live in poverty [17]. Town 3 aimed to provide healthy food for Students in Transition (i.e., youth who are homeless, couch surfing, or living in their cars) by constructing a Little Free Pantry, a freestanding cupboard-like structure where people can take or leave healthy food. See Supplementary Figure S5 for the Little Free Pantry design.

During the interview sessions, leaders noted that they enjoyed participating in eHEART and would recommend the program to other Extension colleagues. They emphasized positive aspects of the eHEART website, including clear guidance on facilitating curriculum meetings and examples of previous initiatives to help groups brainstorm potential projects. Leaders also appreciated the community-driven nature of eHEART projects, which fostered ownership and commitment among group members. 

Several challenging aspects of the eHEART program were discussed. One leader felt that the flexible nature of the eHEART curriculum was not adequately conveyed through the website, which appeared too structured. In practice, most leaders adapted and restructured eHEART meeting activities to best fit group members’ needs. Leaders also reported issues conducting the walkabout due to inclement winter weather and noted that projects required a longer time investment than anticipated (9 months on average).

Recommendations for improvement included providing more guidance on the website for evaluating eHEART projects and contacting stakeholders. One leader suggested adding “mock interviews” to boost members’ confidence in reaching out to local leaders and other community groups. Leaders also suggested creating a one-page checklist outlining all curriculum activities and leveraging the web-based format to include online scheduling tools or project task lists. Table 5 presents supporting quotes for the key findings summarized above.

## 4. Discussion

The eHEART program offers a step-wise approach for local residents to assess their community, identify an issue, develop action plans, and collectively work towards creating positive environmental change. Using a web-based dissemination platform, local health educators successfully engaged rural community members in improving their food or physical activity environments. All three eHEART groups met their project goals and outlined plans to continue their work, highlighting the sustainability of this civic engagement approach.

Strengths of this study include multi-state implementation, recruitment of existing community groups, and adaptation to an online dissemination platform. Implementing the eHEART program across three geographically distinct communities improves overall relevance and generalizability to other rural underserved settings. By recruiting existing community groups, members were more likely to develop a shared sense of purpose and commit to achieving project goals. Lastly, feedback on the eHEART website was predominately positive; leaders found it easy to navigate the website and access facilitation resources. Curriculum modifications such as providing example community projects were well received and contributed to successful implementation. 

The eHEART website was primarily used as training tool for leaders, rather than to directly facilitate curriculum meetings. Modifications to improve website functionality could include fillable PDF worksheets, online group cohesion activities, and discussion boards for communication within and between eHEART groups. As suggested by leaders, the website should convey flexibility in facilitating eHEART meetings (i.e., combining or modifying meeting topics as needed). Further improvements include integrating project management tools to assign tasks and set timelines for completion. 

Additional limitations include the small sample size, selection process for eHEART leaders and participants, and lack of website usage metrics. Only three of nine community groups that applied for the eHEART program were successfully enrolled and completed the process. Strategies for increasing enrollment include clarifying program objectives and providing example eHEART programs on the application form. Inviting educators who were connected with the research team to participate in the eHEART program may have introduced selection bias. However, we were able to enroll a diverse group of communities, which provides support for this dissemination strategy. Lastly, we were unable to measure how often the eHEART website was used by leaders and group members. Tracking the number of visits and time spent on the website would help assess usability and inform design modifications in future studies.

Findings from this work have important implications for creating healthier rural environments through civic engagement to support changes in health behavior. Participation in civic engagement programs may also affect behavior change by fostering self-efficacy and enhancing social support, which are associated with positive health behaviors. [5,9] The relatively narrow scope of the present study limited our ability to assess individual and population-level changes in health behaviors as a result of eHEART projects. However, this remains an important area for future research.

This work, combined with the research team’s prior civic engagement studies, yielded a recently funded National Institutes of Health (NIH) study—a community-randomized controlled trial and process evaluation of this web-based civic engagement approach across ten rural towns in two states. The study will evaluate changes in health behaviors and outcomes among participants, their social networks, and other community residents to understand the impact of this approach to improve rural community health.

## 5. Conclusions

Rural health educators and community organizations can feasibly use the eHEART curriculum to foster environmental changes that support healthy eating and active living. Future studies should consider website modifications to enhance functionality and evaluate the potential behavioral impacts of eHEART projects in rural settings. 

## Figures and Tables

**Table 1:** Overview of the Healthy Eating and Activity in Rural Towns (eHEART) Club curriculum.

Meeting	Objective	Activities	Example Project
1	Identify focus area	Assessing the community (walkabout, debriefing) Selecting an issue (brainstorming, voting) Policy, engagement, and advocacy	Conducted community walkabout around town center Identified need for community physical activity opportunities
2	Develop a purpose	Mapping personal and community assets Developing a purpose statement Identifying and contacting stakeholders	Purpose: Increase physical activity year-round of all individuals in the community Formed partnerships with local Village Board, school district, and other community groups
3	Develop an action plan	True Colors Personality Test Establishing benchmarks Developing an action plan	Planned to install new play equipment and fitness stations at unused local playground Developed fundraising plan Assigned roles and timelines
4	Create a unified message	Group cohesion activity Creating a unified message Creating an eHEART charter Planning for continuation	Created guidelines for meetings and recruiting new members Formed a playground planning committee Established monthly meeting schedule

**Table 2:** eHEART website components.

Section	Content
Home	Introduction to the eHEART program (whiteboard video) Overview of eHEART process and participation benefits Links: “How-To Guide” for navigating the website PDF copy of full eHEART curriculum eHEART application form and guidelines
Meetings	Facilitation guidelines for eHEART meetings 1 to 4 Meeting agenda and materials needed Link to meeting curriculum Meeting overview (whiteboard video) Talking points for each meeting topic/activity Links to handouts/worksheets Action items for next meeting
Resources	Tips for effective leadership and identifying stakeholders Links to external websites for additional information
Example Projects	Brief descriptions of previous HEART Club projects Links to detailed summaries for each project Descriptions of other community health initiatives
Contact Us	Contact information for program staff Form to submit questions about the website or program

**Table 3:** Sociodemographic characteristics of eHEART participants at baseline.

Characteristic (%)	Town 1 (n=5)	Town 2 (n=6)
Age, y (mean [range])	49 (28–55)	46 (29–56)
Sex		
Female	80%	100%
Male	20%	0%
Employment		
Employed full time	100%	83%
Employed part time	0%	17%
Race/ethnicity Non-Hispanic white	100%	100%
Relationship status		
Married	60%	83%
Single	40%	17%
Education		
Master’s degree or higher	20%	17%
Bachelor’s degree	20%	0%
Associate’s degree	40%	33%
High school degree or GED	20%	50%

Note: Sociodemographic characteristics are not presented for the third eHEART group, as it was composed primarily of students and demographic data were only collected for adults.

**Table 4:** Summary of eHEART group progress.

Site	No. of Members	No. of Meetings	Project(s)	Progress	Future Plans
Town 1	4 to 6	6	Develop signage and brochures highlighting existing trail routes	Designed signs to identify five community trails Worked with a local health coalition to create brochures	Publicize trail signs in the town newspaper Host a celebratory community walk on the trails
Town 2	6 to 8	5	Improve active recess opportunities Schedule recess before lunch breaks Restructure cafeteria to encourage healthy food choices	Created new outdoor play areas and indoor activity wall Campaigned for recess before lunch Improved cafeteria layout Formed a teacher-led school wellness committee	Create an indoor walking path Continue wellness committee initiatives to improve food and physical activity options
Town 3	4 to 6	6	Construct tiny food pantries to provide healthy food for students in need	Secured donations for building supplies and signage Built first food pantry (Little Free Pantry)	Build a second pantry Weather-proof pantries Partner with 4-H Council to maintain pantries as a service project

Note: 4-H is a national youth development organization in the United States.

**Table 5:** Qualitative feedback from eHEART leaders.

Category	Selected Quotes
Positive aspects	“This was a great tool to spearhead some movement at the school. I would love to train all the UW Extension educators in WI to do the same!” (Town 2) “Having examples of what other people had done was beneficial and helped with initial project brainstorming.” (Town 1) “Another strength (of group members) is the perseverance to see this project through and the commitment to making it sustainable.” (Town 3)
Challenging aspects	“Appreciated having flexibility (in facilitating meetings), which isn’t conveyed from the website as it appears inflexible.” (Town 3) “The walkabout did not work for us. The trails were all over town and since it was winter time, it was always dark when our group met.” (Town 1)
Recommendations for improvement	“Give guidance or doing role playing to boost confidence for group members that aren’t comfortable talking with stakeholders.” (Town 1) “Checklist like a one-pager overall view for an entire year of what to do. For example: Meeting 1, do this between meetings, Meeting 2 and so on.” (Town 2)

## Supplementary Materials

The following are available online at https://www.mdpi.com/1660-4601/17/7/2571/s1, Figure S1: eHEART website screenshots, Figure S2: Project timeline for eHEART leaders, Figure S3: Town 1 eHEART trail maps, Figure S4: Town 2 eHEART indoor and outdoor recess projects, and Figure S5: eHEART Little Free Pantry.

## References

[1] Patterson P.D., Moore C., Probst J., Shinogle J. (2004). Obesity and physical inactivity in rural America. J. Rural Health.

[2] Trivedi T., Liu J., Probst J., Merchant A., Jones S., Martin A. (2015). Obesity and obesity-related behaviors among rural and urban adults in the USA. Rural Remote Health.

[3] Chenarides L., Jaenicke E.C. (2018). Documenting the link between poor food access and less healthy product assortment across the US. Appl. Econ. Perspect. Policy.

[4] Quinn C., Slater S., Barker D., Chaloupka F. (2015). Availability of Local Public Recreational Facilities and Programs for Physical Activity. A BTG Research Brief.

[5] Brown A.G.M., Hudson L., Chui K., Metayer N., Lebron-Torres N., Seguin R., Folta S. (2017). Improving heart health among Black/African American women using civic engagement: A pilot study. BMC Public Health.

[6] Cohen D.A., Han B., Derose K., Williamson S., Marsh T., McKenzie T. (2013). Physical activity in parks: A randomized controlled trial using community engagement. Am. J. Prev. Med..

[7] Seguin R.A., Folta S., Sehlke M., Nelson M., Heidkamp-Young E., Fenton M., Junot B. (2014). The StrongWomen Change Clubs: Engaging residents to catalyze positive change in food and physical activity environments. J. Environ. Public Health.

[8] Seguin R.A., Paul L., Folta S., Nelson M., Strogatz D., Graham M., Diffenderfer A., Eldridge G., Parry S. (2018). Strong Hearts, Healthy Communities: A community-based randomized trial for rural women. Obesity.

[9] Seguin R.A., Sriram U., Connor L., Silver A., Niu B., Bartholomew A. (2018). A civic engagement approach to encourage healthy eating and active living in rural towns: The HEART Club pilot project. Am. J. Health Promot..

[10] Slater S., Pugach O., Lin W., Bontu A. (2016). If you build it will they come? Does involving community groups in playground renovations affect park utilization and physical activity?. Environ. Behav..

[11] Seguin R., Economos C., Nelson M., Hyatt R., Palombo R., Reed P. (2008). Design and national dissemination of the StrongWomen Community Strength Training Program. Prev. Chronic Dis..

[12] DataUSA. https://datausa.io/profile/geo/hardin-mt.

[13] DataUSA. https://datausa.io/profile/geo/clinton-wi.

[14] Bergman E.A., Buergel N.S., Englund T.F., Femrite A. (2004). The relationship of meal and recess schedules to plate waste in elementary schools. J. Child Nutr. Manag..

[15] Tanaka C., Richards K.L., Takeuchi L.S., Otani M., Maddock J. (2005). Modifying the recess before lunch program. Calif. J. Health Promot..

[16] Montana Office of Public Instruction (2003). A Recess before Lunch Policy Implementation Guide.

[17] DataUSA. https://datausa.io/profile/geo/nikiski-ak.

