# Supplementary material for: Web-Based Dissemination of a Civic Engagement Curriculum to Promote Healthy Eating and Active Living in Rural Towns: The eHEART Study
Source: Int J Environ Res Public Health. 2020 Apr 9;17(7):2571. doi: 10.3390/ijerph17072571 (PMC7177878; doi:10.3390/ijerph17072571)
Supplement: Supplementary file 1 [file ijerph-17-02571-s001.pdf]

Supplementary Figure S1. eHEART Website Screenshots.

Supplementary Figure S2. Project Timeline for eHEART Leaders.

Supplementary Figure S3. Town 1 eHEART Trail Maps.

Supplementary Figure S4. Town 2 eHEART Indoor and Outdoor Recess Projects.

Supplementary Figure S5. Town 3 eHEART Little Free Pantry.

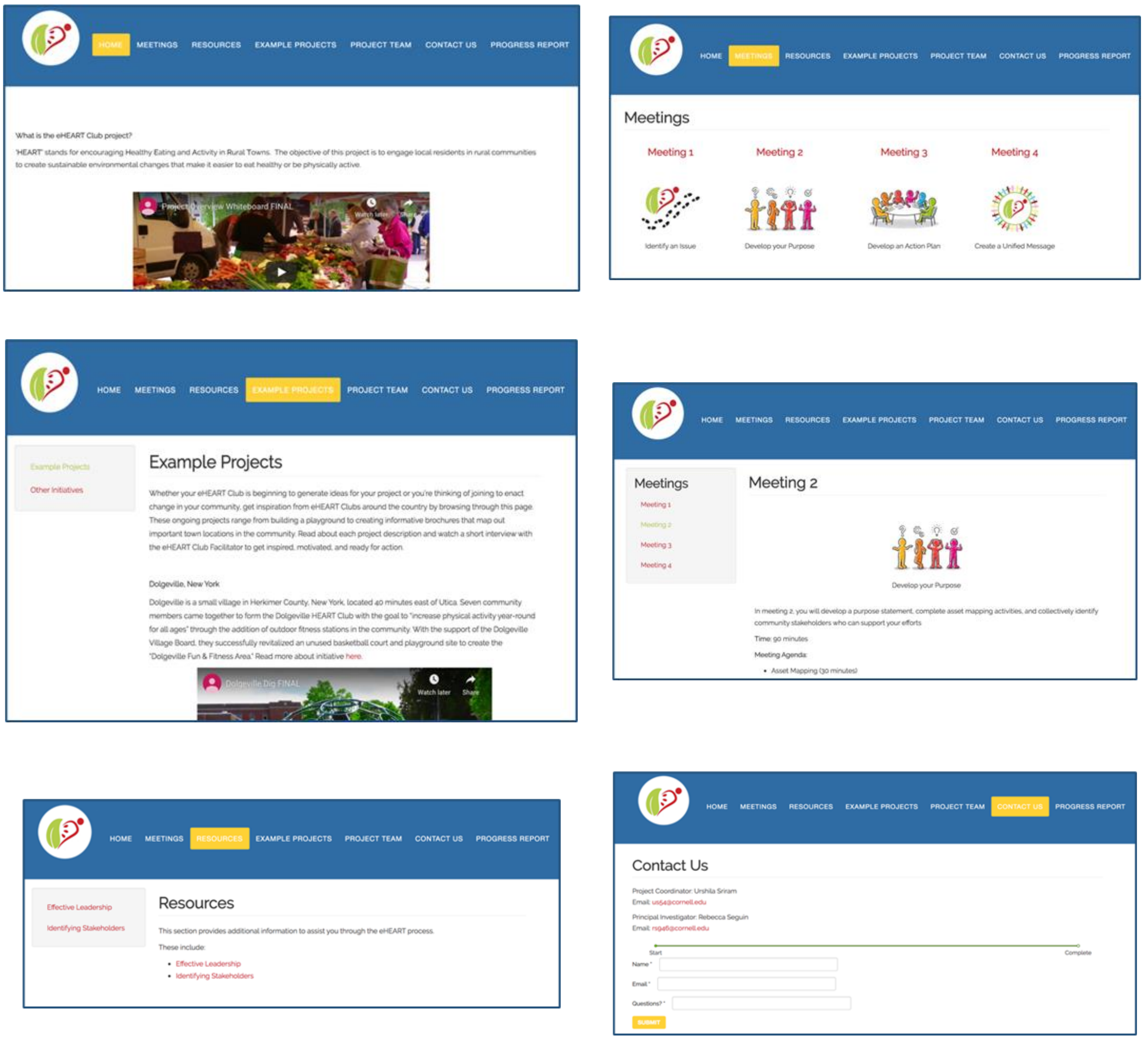

Figure S1. eHEART Website Screenshots.

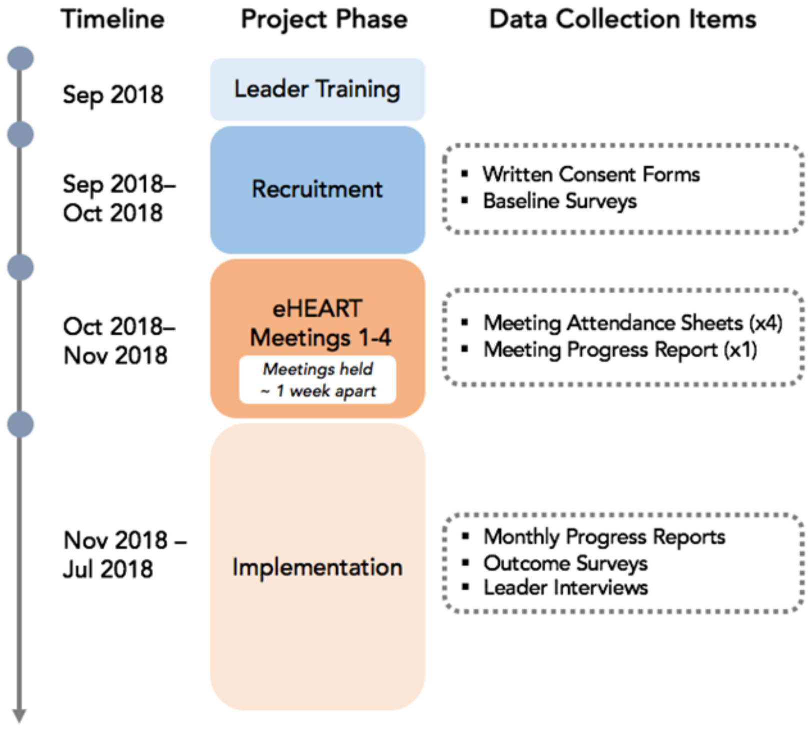

Figure S2. Project Timeline for eHEART Leaders.

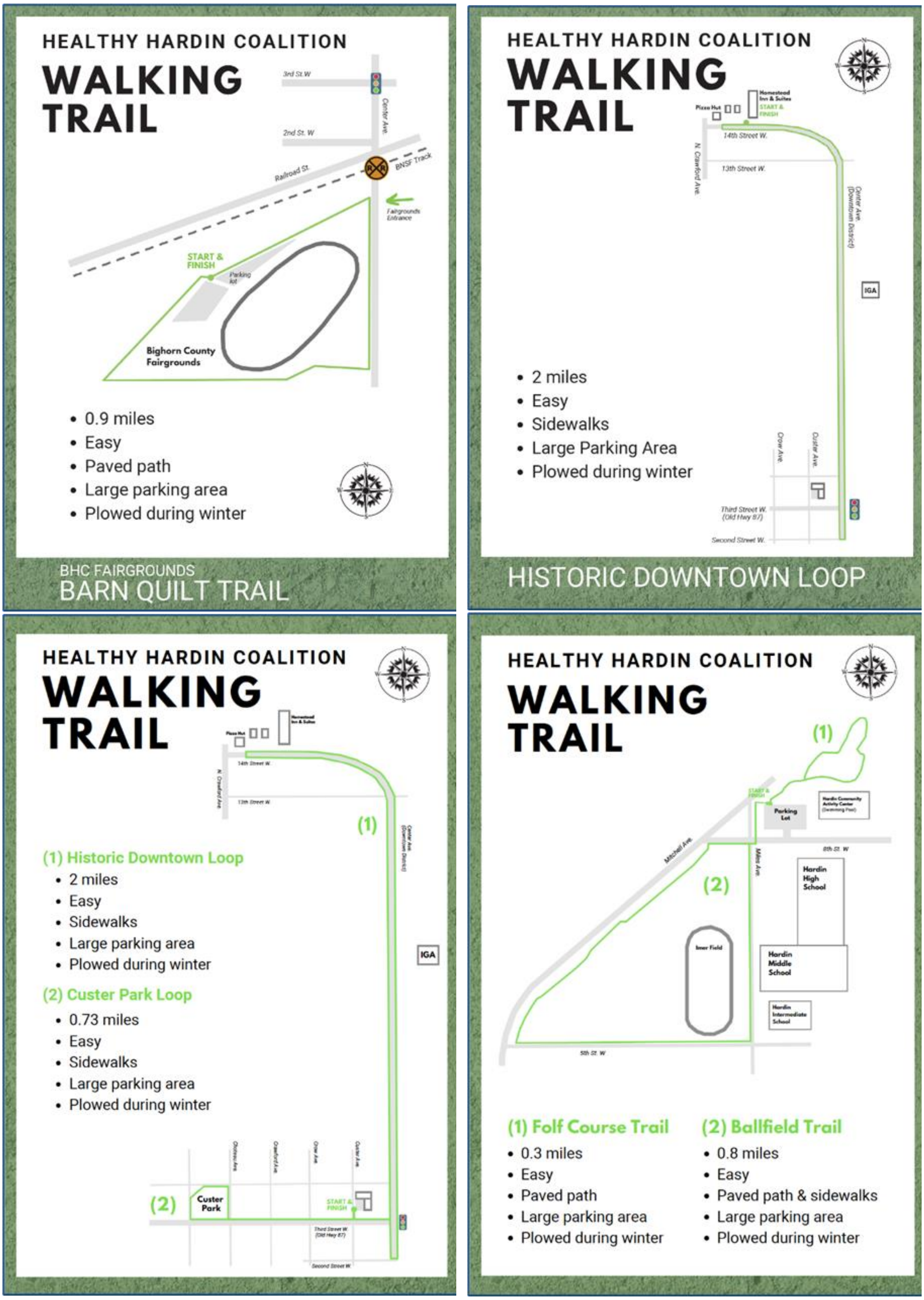

Figure S3. Town 1 eHEART Trail Maps.

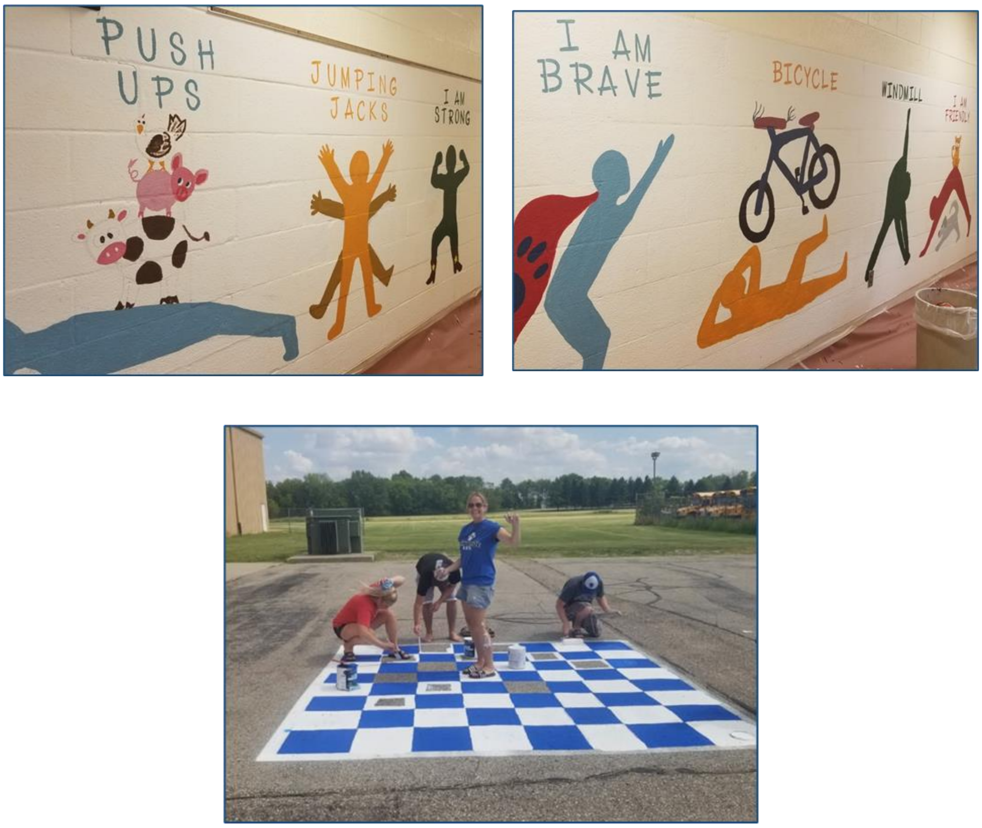

Figure S4. Town 2 eHEART Indoor and Outdoor Recess Projects.

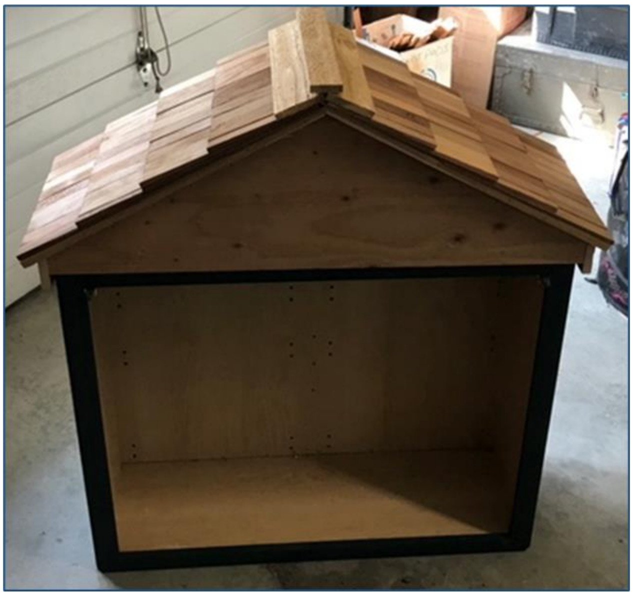

Figure S5. Town 3 eHEART Little Free Pantry.
